# Patterns in Genotype Composition of Indian Isolates of the Bombyx mori Nucleopolyhedrovirus and Bombyx mori Bidensovirus

**DOI:** 10.3390/v13050901

**Published:** 2021-05-13

**Authors:** Mudasir Gani, Sergei Senger, Satish Lokanath, Pawan Saini, Kamlesh Bali, Rakesh Gupta, Vankadara Sivaprasad, Johannes A. Jehle, Jörg T. Wennmann

**Affiliations:** 1 Division of Entomology, Faculty of Agriculture, Sher-e-Kashmir University of Agricultural Sciences & Technology, Kashmir 193 201, J&K, India; mudasir32@gmail.com; 2 Julius Kühn Institute (JKI)-Federal Research Centre for Cultivated Plants, Institute for Biological Control, Heinrichstr. 243, 64287 Darmstadt, Germany; sergei.senger@web.de (S.S.); johannes.jehle@julius-kuehn.de (J.A.J.); 3 Central Sericultural Research & Training Institute, Central Silk Board, Ministry of Textiles, Government of India, Srirampura, Mysore 570 008, Karnataka, India; satigene@gmail.com (S.L.); siva.nsso@gmail.com (V.S.); 4 Central Sericultural Research & Training Institute, Central Silk Board, Ministry of Textiles, Government of India, Pampore, Kashmir 192 121, J&K, India; saini.pawansaini.pawan5@gmail.com; 5 Division of Entomology, Sher-e-Kashmir University of Agricultural Sciences and Technology, Chatha, Jammu 180 009, J&K, India; balikamlesh76@gmail.com (K.B.); rkguptaentoskuast@gmail.com (R.G.)

**Keywords:** *Bombyx mori*, silkworm, *Baculoviridae*, *Bidnaviridae*, baculovirus, densovirus, genotype, genetic variation, single nucleotide polymorphism, population structure

## Abstract

The mulberry silkworm, *Bombyx mori* (L.), is a model organism of lepidopteran insects with high economic importance. The viral diseases of the silkworm caused by Bombyx mori nucleopolyhedrovirus (BmNPV) and Bombyx mori bidensovirus (BmBDV) inflict huge economic losses and significantly impact the sericulture industry of India and other countries. To understand the distribution of Indian isolates of the BmNPV and to investigate their genetic composition, an in-depth population structure analysis was conducted using comprehensive and newly developed genomic analysis methods. The seven new Indian BmNPV isolates from Anantapur, Dehradun, Ghumarwin, Jammu, Kashmir, Mysore and Salem grouped in the BmNPV clade, and are most closely related to Autographa californica multiple nucleopolyhedrovirus and Rachiplusia ou multiple nucleopolyhedrovirus on the basis of gene sequencing and phylogenetic analyses of the partial *polh*, *lef-8* and *lef-9* gene fragments. The whole genome sequencing of three Indian BmNPV isolates from Mysore (-My), Jammu (-Ja) and Dehradun (-De) was conducted, and intra-isolate genetic variability was analyzed on the basis of variable SNP positions and the frequencies of alternative nucleotides. The results revealed that the BmNPV-De and BmNPV-Ja isolates are highly similar in their genotypic composition, whereas the population structure of BmNPV-My appeared rather pure and homogenous, with almost no or few genetic variations. The BmNPV-De and BmNPV-Ja samples further contained a significant amount of BmBDV belonging to the *Bidnaviridae* family. We elucidated the genotype composition within Indian BmNPV and BmBDV isolates, and the results presented have broad implications for our understanding of the genetic diversity and evolution of BmNPV and co-occurring BmBDV isolates.

## 1. Introduction

The silk moth, *Bombyx mori* (L.), is an economically and culturally important insect that is domesticated for silk production, mainly in China and India [1]. Domestic mass rearing of silk moth is highly labor-intensive and usually handled by local farmers, who continuously rear larvae from hatching to pupation with the only available food source, the leaves of the mulberry plant [2,3]. The pupating larvae produce cocoons from which silk thread can be harvested. The major constraints for increased cocoon yield in sericulture are the occurrence of diseases such as grasserie, flacherie, muscardine and pebrine caused by virus, bacteria, fungi and microsporidia infection, respectively [4,5,6]. Viruses are considered the most important pathogens in silk moth rearing, causing serious and significant economic damage, and thus posing a serious threat to global sericulture [7]. Silkworm losses due to diseases in India are about 15–20%, of which more than 50% are attributed to viral infection only [8]. 

The grasserie disease of silkworm caused by the Bombyx mori nucleopolyhedrovirus (BmNPV) (genus *Alphabaculovirus*, family *Baculoviridae*) is the most abundant and well-described pathogen in the history of the silk industry, and has a high impact on the sericulture agrosystem in India and worldwide [9,10]. BmNPV infects larval stages of the silk moth, and symptoms of a BmNPV infection include shining and fragile skin, swollen inter-segmental regions, appearance of milky white haemolymph, death and the hanging of larvae by their prolegs [11,12]. 

The *Bombyx mori nucleopolyhedrovirus* species comprises isolates of the BmNPV and Bombyx mandarina nucleopolyhedrovirus (BomaNPV) [13,14,15]. BmNPV and BomaNPV isolates have a circular closed dsDNA genome which is between 125,437 and 129,646 bp long and encodes between 127 and 143 open reading frames (ORFs) [10,16]. At least 12 different BmNPV and BomaNPV isolates were entirely sequenced including isolates from the major silk producing countries of China, India, Brazil, Japan and Korea [16,17,18,19]. To date, only one isolate from India (BmNPV-India) was entirely sequenced and characterized, with a genome size of 126,879 bp and 138 ORFs [18]. Isolates of BmNPV were found as the most serious and dominant viral pathogen in almost all the sericultural practicing areas, with a maximum prevalence of 17.32% in Ghumarwin, Himachal Pradesh, and an overall average infection percentage of 7.19% in India during 2017 [6]. 

Another important viral pathogen that was described specifically in silk moth larvae, is the Bombyx mori bidensovirus (BmBDV) (genus *Bidensovirus*, family *Bidnaviridae*), a small linear ssDNA virus with a bipartite (VD1 = 6 kb; VD2 = 6.5 kb) genome [20]. BmBDV causes viral diarrhea-like symptoms in infected larvae and poses, besides BmNPV, a second major threat to the sericulture industry. Similar to BmNPV, isolates of BmBDV were characterized, including the deciphering of their entire genome sequence, from China, Japan and India [21,22,23]. 

Thus far, whole genome analysis of BmNPV/BomaNPV and BmBDV allowed the establishment of consensus genomes of these viruses that reflect the entity of the sequenced virus populations. Recent analyses focusing on the population structure of baculoviruses showed that the genotypic composition of single virus isolates can be rather complex [24,25,26]. Genetic diversity was determined by detecting single nucleotide polymorphisms (SNPs) and the frequencies of alternative occurring nucleotide variants in these positions, which allowed for the differentiation of homogeneous, heterogeneous and mixed isolates [24,25,26].

An in-depth population structure analysis of BmNPV and BmBDV isolates has not been conducted, but it offers us the possibility to understand the distribution of different isolates and to investigate their genetic composition. Here, we present a genomic analysis of various geographic BmNPV samples from India, which also reveals the presence of BmBDV. Three new BmNPV samples were sequenced and genetic diversity was determined using variable SNP positions across the genotype population. We provide a comprehensive analysis of BmNPV and BmBDV isolates from three different regions in India, namely Mysore, Jammu and Dehradun, which led to the identification of mixed and heterogeneous BmNPV and BmBDV isolates, thus allowing for a more detailed picture of the composition and spatial distribution of genotypes within the BmNPV and BmBDV virus groups.

## 2. Materials and Methods

### 2.1. Insects and Viruses

**Figure 1:**
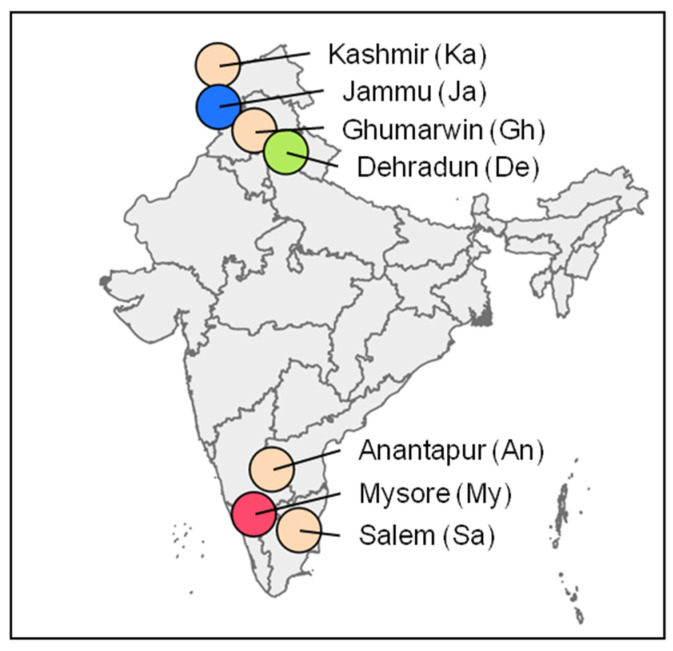
BmNPV occlusion body stocks collected from different locations in India. Stock abbreviations are written in brackets.

The virus samples used in this study were isolated from naturally field-infected *B. mori* larvae collected from different climatic regions of India: the temperate region of Kashmir (Ka), the subtropical areas of Jammu (Ja), Ghumarwin in Himachal Pradesh (Gh) and Dehradun in Uttarakhand (De), as well as the tropical locations of Mysore in Karnataka (My), Anantapur in Andhra Pradesh (An) and Salem in Tamil Nadu (Sa) (Figure 1, Table 1) [6]. Although the prevalence of BmNPV was found throughout the sericulture practicing areas of India, the selected locations were hot spots, where a heavy incidence of BmNPV was observed or reported. The extraction, purification and standardization of baculovirus occlusion bodies (OBs) was performed as described in Liang et al. [27], with some modifications. In brief, *B. mori* larvae with symptoms of baculovirus infection, such as swollen intersegmental regions and liquification of the body, were collected individually in glass vials. The larval samples were combined with 1 mL 0.1% sodium dodecyl sulphate (SDS) and the cadavers were disrupted by using a pestle followed by vortexing for 2 min and filtration through three-layers muslin cloth. The suspension was centrifuged briefly at 100× *g* for 10 s to settle larger larval debris. The supernatant was carefully removed to a new centrifuge tube while the pellet was processed twice with an additional 0.1% SDS and above mentioned centrifugation steps. Then, all supernatants were combined and centrifuged at 15,000× *g* for 5 min using a Remi R-8C (Remi Group, Mumbai, India) centrifuge. The pellet was re-suspended in 1 mL distilled water and BmNPV OB samples from larvae of each location were named according to their sampling sites (Table 1). For the molecular studies, the genome sequence of BmNPV-India (GenBank accession no. JQ991010) was included [18]. This isolate originated from Nagpur (Central India) and was plaque purified in cell culture prior to its genome sequencing [18].

### 2.2. Extraction of Viral DNA

For PCR and whole genome analysis, the viral DNA of each BmNPV isolate was isolated following standard laboratory protocols [28]. In brief, the OB matrix was solubilized in 0.1 M Na_2_CO_3_ for 1 h, followed by adjusting the pH to 7 using 1 M HCl. The samples were treated with RNaseA (90 µg/mL) at 37 °C for 10 min, followed by incubation with proteinase K (250 µg/mL) and 1% SDS at 50 °C for 60 min. Protein debris and DNA were separated by phenol/chloroform/isoamylalcohol (25:24:1, v:v:v) extraction, and DNA precipitation was performed with ethanol at −20 °C overnight [29]. The DNA pellet was dissolved in ultra-pure water and the DNA concentration and purity were determined with a NanoDrop 2000c (Thermo Fisher Scientific, Waltham, MA, USA) spectrophotometer.

### 2.3. Partial Gene Analysis

Partial gene fragments of *polyhedrin* (*polh*), the *late expression factor 8* (*lef-8*) and the *late expression factor 9* (*lef-9*), common marker genes for baculovirus identification and species demarcation, were amplified by polymerase chain reaction (PCR) using the degenerated primers prPH-1/2 (prPH-1B), prL8-1/2 (prL8-1B) and prL9-1/2, respectively [14,15,30]. The gene fragments were amplified in 50 µL containing 2 µL 10 mM dNTP, 1 µL of each primer (10 µM), 5 µL 10× reaction buffer, including MgCl_2_, 0.5 µL 1.0 U Taq DNA polymerase and 2.5 µL of genomic DNA (adjusted to 25 ng/µL). PCR conditions were set as described in Jehle et al. [14]. Success of the PCR reaction was checked visually by agarose gel electrophoresis. After all samples were detected positive for containing *polh*, *lef-8* and *lef-9* fragments, the PCR products were purified from the PCR reaction mixture by using the Zymo PCR/concentrator kit (Zymo Research Europe GmbH, Freiburg, Germany), followed by Sanger sequencing using the forward and reverse primers (StarSEQ GmbH, Mainz, Germany) [14]. Raw forward and reverse sequences of each sample were quality trimmed on both ends and merged in overlapping regions by mapping using Geneious Prime v11.0.6 (Biomatters Ltd., Auckland, New Zealand). The Sanger chromatograms were checked manually for ambiguities. The consensus sequence was extracted and its *polh*, *lef-8* or *lef-9* identity was verified by Blast search. The final partial *polh*, *lef-8* and *lef-9* sequences of each BmNPV isolate were uploaded to GenBank (Table 1). 

The nucleotide sequences of the partial three genes were aligned individually using MAFFT v7.450, as implemented in Geneious Prime v11.0.6. The alignments included the partial sequences of further alphabaculoviruses [15]. The three alignment files were concatenated to a single alignment file. The nucleotide alignment was used for maximum likelihood (ML) phylogenetic analysis (500 bootstrap replicates) in MEGA v7 [31].

About 100 to 200 ng purified genomic DNA of the BmNPV samples from My, De and Ja (Figure 1, Table 2) were subjected to whole genome sequencing on an Illumina NextSeq500 platform (StarSEQ GmbH, Mainz, Germany), with a targeted total amount of 2.5 million paired-end reads of 151 nt in length (Table 2). All bioinformatic steps were conducted on a Galaxy server of the Julius Kühn Institute. Reads were quality filtered and adapter trimmed using Trim Galore v0.6.3 [32], with a quality (Phred) score >30 and a minimum read length of 50 and 51 nt for paired and un-paired reads, respectively (Table 2). Each sequenced BmNPV sample was subjected to a de novo assembly of reads using CLC de novo assembly tool v1.0.0 (Qiagen GmbH, Hilden, Germany), with a minimum contig length of 5000 nt. From the BmNPV-My sample, a complete BmNPV genome sequence, termed BmNPV-My, of 127,582 bp could be assembled. For the BmNPV-De and BmNPV-Ja samples, the assembly of contigs encompassing the entire BmNPV genome was not successful. The genome sequence of BmNPV-My was annotated by transferring annotations from BmNPV-India (JQ991010) with a similarity in nucleotide sequence >95%, as implemented in Geneious Prime v11.0.6. Annotations were checked by hand and adjusted if necessary. Missing open reading frames (ORF) that were at least 150 nt in length and did not overlap by more than 50 nt with other ORFs were added if needed. The reliability of the BmNPV-My consensus sequence was tested by mapping the quality filtered reads using BWA-MEM v0.8.0 [33] to check the coverage of the genome. The annotated BmNPV-My genome was uploaded and published on GenBank (MW842985).

### 2.4. Whole Genome Sequencing

A blast search of de novo assembled contigs that were obtained for the three sequenced BmNPV samples resulted in the discovery of two entire bidensovirus genome molecules, VD1 (6542 nt) and VD2 (6002 nt), within the BmNPV-Ja sample. No entire VD1 and VD2 encompassing contigs were obtained from the de novo assembly of BmNPV-De and BmNPV-My reads. ORFs with a start (ATG) and stop (TAA/TGA/TAG) were annotated for VD1 and VD2 of the BmNPV-Ja sample, and the two consensus sequences of VD1 and VD2 were checked by the assembling of reads using BWA-MEM v0.8.0 [33]. The genome molecules VD1 and VD2 were termed BmBDV-Ja VD1 and BmBDV-Ja VD2, respectively, and published on GenBank (MW842986 and MW842987).

### 2.5. SNP Genotyping of BmNPV and BmBDV Isolates

To decipher the genetic composition of the sequenced BmNPV-De, BmNPV-Ja and BmNPV-My samples, the entity of quality filtered reads were mapped separately against the reference genome sequences of BmNPV-My (MW842985) and BmNPV-India (JQ991010) using BWA-MEM v0.8.0 [33]. The alignment files that were generated for each reference sequence were used for the detection of SNP positions using MPileup v2.1.1 [34,35]. Variant sites only were kept by using BCFTools v1.0 [36], resulting in 1723 and 1785 variable SNP positions for the BmNPV-My and BmNPV-India reference-based analysis, respectively. Variable SNP positions were analyzed quantitatively and qualitatively by using the bacsnp v0.1.4 package in R (R v4.0.3 in RStudio v1.1.443) [37].

To quantify the number of BmBDV reads within the sequenced BmNPV-Ja, BmNPV-De and BmNPV-My sample, the entities of reads were mapped against the reference sequence of BmBDV-Ja. For this, both molecules (VD1 = 6542 nt; VD2 = 6002 nt) were concatenated with a random 300 nt spacer (Figure S1), resulting in a single BmBDV-Ja reference sequence of 12,845 nt in length (Figure S1). The quality filtered reads of the BmBDV-De, BmBDV-Ja and BmNPV-My sample were mapped against this artificial BmBDV-Ja VD1 + VD2 reference genome. Reads did not map against the 300 nt spacer between VD1 and VD2 (Figure S2). The number of reads that mapped against VD1 and VD2 were quantified from the BmNPV-De, BmNPV-Ja and BmNPV-My mapping files, and were used for an estimation of VD1 to VD2 ratio by using the read per kilo base per million reads (RPKM) method. The mapping files were further used for the detection of variable SNP positions following the same workflow used for the BmNPV described above [37].

### 2.6. Genome-Based Phylogeny

The collinear genomes of various BmNPV isolates and Autographa californica multiple nucleopolyhedrovirus isolate C6 (AcMNPV-C6) (L22858) were aligned using progressive Mauve algorithm v1.1.1 in Geneious Prime v11.0.6. For the BmBDV genome alignment, both genome molecules VD1 and VD2 of already-published isolates were extracted from GenBank. The nucleotide sequences of VD1 and VD2 were concatenated with a random 300 nt spacer (Figure S1) between them, and the concatenated BmBDV genomes were aligned using MAFFT v7.450, as implemented in Geneious Prime v11.0.6. Based on the BmNPV and BmBDV whole genome alignments, phylogenetic trees were constructed using maximum likelihood algorithm, with Best-Fit substation model implemented in MEGA v7 [31].

## 3. Results

### 3.1. Genotype Composition of BmNPV Isolates

**Figure 2:**
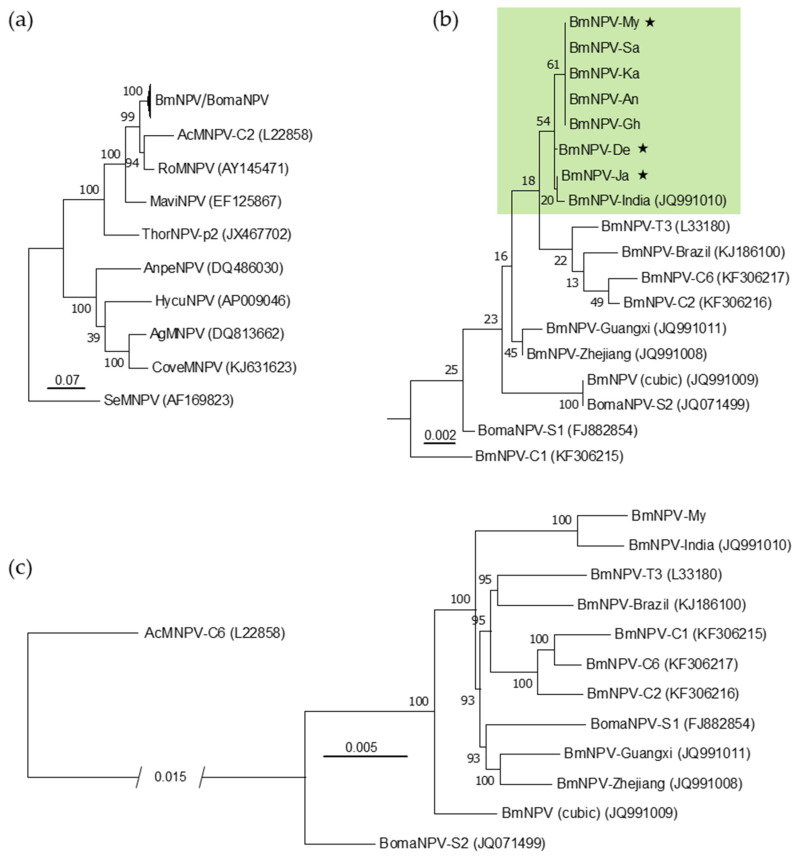
Maximum likelihood (ML) tree based on concatenated partial nucleotide sequences of *polh*, *lef-8* and *lef-9* of selected alphabaculoviruses (group I, clade Ia) [14,15]: (**a**) Various BmNPV and Bombyx mandarina nucleopolyhedrovirus (BomaNPV) isolates, Autographa californica nucleopolyhedrovirus C2 (AcMNPV-C2), Rachiplusia ou multiple nucleopolyhedrovirus (RoMNPV), Maruca vitrata nucleopolyhedrovirus (MaviNPV); Thysanopolusia orichalcea nucleopolyhedrovirus (ThorNPV), Antheraea pernyi nucleopolyhedrovirus (AnpeNPV), Hyphantria cunea nucleopolyhedrovirus (HycuNPV), Anticarsia gemmatalis multiple nucleopolyhedrovirus (AgMNPV), Condylorrhiza vestigialis nucleopolyhedrovirus (CoveNPV) and Spodoptera exigua multiple nucleopolyhedrovirus (SeMNPV). SeMNPV was set as outgroup; (**b**) Detailed view on the BmNPV and BomaNPV clade. Isolates originated from India are highlighted in green. The three isolates that were conducted for whole genome sequencing are marked by a star; (**c**) ML analysis based on a whole genome nucleotide alignment of the collinear genomes of isolates of the BmNPV and BomaNPV clade, as well as AcMNPV that served as outgroup. The robustness of the phylogenetic analysis was tested with 500 bootstrap replicates. GenBank accession numbers are given in brackets behind isolates’ names.

PCR amplification of the partial *polh*, *lef-8* and *lef-9* fragments of the seven new Indian BmNPV isolates (Table 1), and the phylogenetic analyses of their concatenated sequences with those of other BmNPV/BomaNPV isolates, as well as members of the genus *Alphabaculovirus* (group I), identified all isolates within the BmNPV/BomaNPV clade; they are all closely related to AcMNPV and the Rachiplusia ou multiple nucleopolyhedrovirus (RoMNPV) (Figure 2a,b). Within the BmNPV/BomaNPV clade, the seven new isolates were not identical in their partial *polh*, *lef-8* and *lef-9* sequences to previously described BmNPV isolates, including the previously described Indian isolate BmNPV-India [18]. Together with BmNPV-India, the new Indian isolates BmNPV-My, -Sa, -Ka, -An, -Gh, -De and -Ja formed a common clade, and were separated from the other Asian and Brazilian (BmNPV-Brazil) isolates (Figure 2b) [10,16]. Based on their partial gene alignment, the sequences of BmNPV-My, -Sa, -Ka, -An, and -Gh were identical, whereas BmNPV-De and -Ja were different to them and between each other, therefore creating a common subclade together with BmNPV-India (Figure 2b). 

Due to the phylogenetic separation within the Indian BmNPV clade and the distance between their geographic origins, the isolates of BmNPV-My, BmNPV-De and BmNPV-Ja (Figure 2b) were selected for whole genome sequencing, which led to the de novo assembly of a BmNPV-My contig of 127,582 bp in length that encompassed the entire circular BmNPV genome. For BmNPV-De and BmNPV-Ja, the de novo assembly of a single contig comprising the entire BmNPV genome was not possible, but it resulted in multiple contigs. Based on the nucleotide alignment of entire BmNPV genomes, which was also used for a more accurate phylogenetic reconstruction than the partial *polh*, *lef-8* and *lef-9* sequences (Figure 2c), the nucleotide sequence identity between BmNPV-My and BmNPV-India was calculated as 98%. BmNPV-My and BmNPV-India generated a common clade according to the alignment of their entire genomes (Figure 2c), confirming the close relatedness of both isolates (Figure 2b,c).

**Figure 3:**
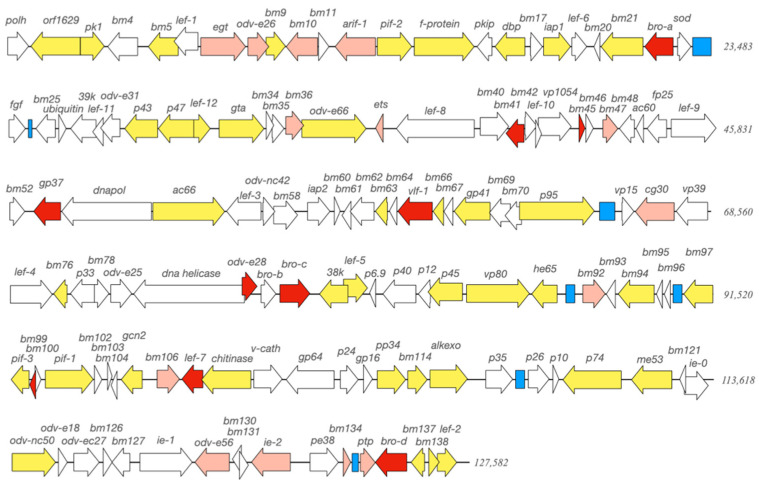
Schematic linearized presentation of the circular covalently closed dsDNA genome of BmNPV-My. Arrows indicate open reading frames (ORFs) and their direction of transcription. Gene names are given above each annotation. Per definition, *polh* (top left) is set as the first ORF in the genome of baculoviruses. Colors of ORFs indicate the similarity in amino acid sequence in comparison to BmNPV-India: white—identical amino acid sequence; yellow—99% to <100% identity; pink—98% to <99% identity; red—<98% identity. Blue boxes indicate homologous repeat regions (*hrs*).

The assembled genome of BmNPV-My isolate had 139 open reading frames (ORF), of which 138 had homologs in BmNPV-India [18]. The additional ORF in BmNPV-My was identified as *bro-b* (Figure 3). Comparing the amino acid sequence identity of all annotated ORFs with the BmNPV-India, 116 ORFs were >99% similar or identical to their counterparts in BmNPV-India, 13 ORFs were <99% to >98% similar and only 10 were less than 98% similar (Figure 3). Among the five least similar amino acid sequences (<97% similarity), three were *bro* genes (*bro-a*, *bro-c* and *bro-d*) and two were hypothetical ORFs (*bm45* and *bm99*) (Figure 3, Table S1). 

**Figure 4:**
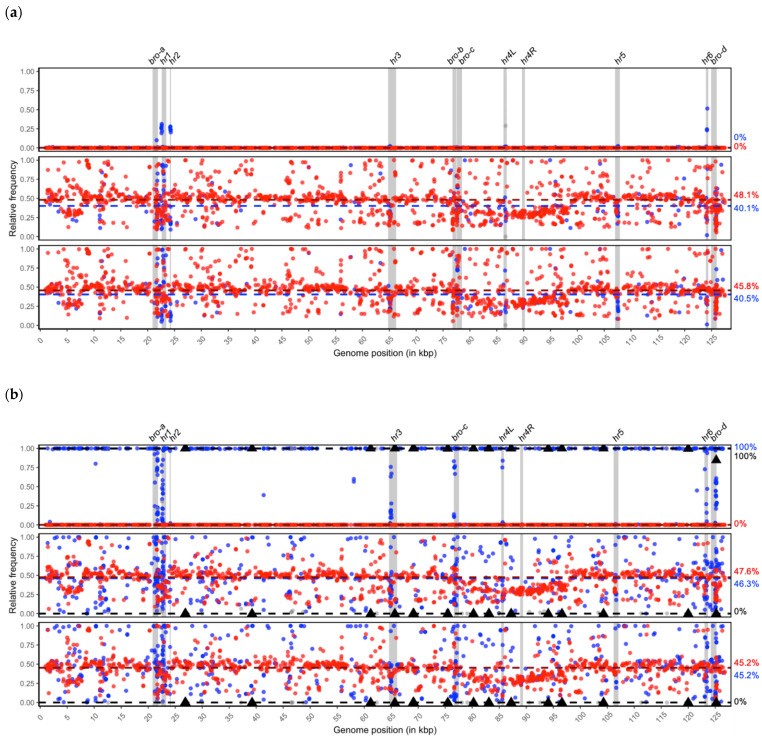
Common reference-based SNP detection method using either (**a**) BmNPV-My or (**b**) BmNPV-India as reference. Analysis of BmNPV-My, BmNPV-De and BmNPV-Ja are given on top, middle and bottom, respectively. Black triangles: solely BmNPV-My specific; red: BmNPV-De and BmNPV-Ja specific; blue: BmNPV-My, BmNPV-De and BmNPV-Ja specific; light grey dots: either BmNPV-My + BmNPV-De or BmNPV-My + BmNPV-Ja specific SNP positions. Median SNP frequencies are presented by black (=BmNPV-My), blue (=BmNPV-De and BmNPV-Ja) and red (=BmNPV-My, BmNPV-De and BmNPV-Ja) dashed lines. Medians are given to the right of the plot in corresponding colors.

To test for genotypic variation within the sequenced samples BmNPV-My, BmNPV-De and BmNPV-Ja, a common reference-based approach for detecting variable SNP positions was followed. In this workflow, the entire set of reads of each sample were mapped individually to a common reference, either BmNPV-My (MW842985) or BmNPV-India (JQ991010), resulting in three alignment files for each sequenced sample and reference. By the use of a common reference, the variable SNP positions within the three samples were linked, which allowed for the determination of SNP position specificities. To quantify the degree of variability of each sample, the reference nucleotide and the alternative occurring nucleotide were counted in each SNP position, allowing a quantitative presentation of the isolate’s genetic variability (Figure 4a,b).

In total, 1709 and 1785 variable SNP positions were detected for the BmNPV-My and BmNPV-India reference-based analysis, respectively (Figure 4a,b). According to the applied bacsnp workflow for SNP specificity determination, 1464 (reference BmNPV-My based) and 1113 (reference BmNPV-India based) SNP positions were identified as specific to the BmNPV-De and BmNPV-Ja (De + Ja) isolates. For all three geographic isolates BmNPV-My, BmNPV-De and BmNPV-Ja (My + De + Ja), 244 and 631 SNP positions were found to be variable, according to the BmNPV-My and BmNPV-India based references, respectively. When BmNPV-India was used as reference, the number of positions that were found to be variable only for isolate BmNPV-My (My) was 18. No solely BmNPV-My specific SNP positions were found in the BmNPV-My reference-based analysis (Figure 4a,b). Additional SNP positions were variable for the combinations of My+De (0 and 22 based on BmNPV-My and BmNPV-India, respectively) and My + Ja (1 and 1 based on BmNPV-My and BmNPV-India, respectively) (Figure 4a,b).

For the self-mapping of BmNPV-My reads against their own BmNPV-My reference consensus sequences, the overall detected SNP frequencies were below 50%, and were only detected to be slightly more variable within repeat regions *bro-a*, *hr1*, *hr2* and *hr6* (Figure 4a, top). In these regions with increased variability, reads can align falsely, causing the detection of alleged SNPs. An overall genotype purity of the BmNPV-My isolate was concluded by the missing frequencies of alternative nucleotides in the detected SNP positions. This was further reflected by the median frequencies of the My + De + Ja (ƒ = 0%) and De+Ja (ƒ = 0%) specific positions (Figure 4a).

For isolate BmNPV-De, the median SNP frequencies of De + Ja and My + De + Ja were ƒ = 48.1% and ƒ = 40.1%, respectively (Figure 4a, middle). Similar median SNP frequencies were measured for isolate BmNPV-Ja, where the De + Ja and My + De + Ja variable SNP positions had alternative nucleotide frequencies of ƒ = 45.8% and ƒ = 40.5%, respectively (Figure 4a, bottom). In comparison to BmNPV-My, the isolates BmNPV-De and BmNPV-Ja appeared more variable in their SNP frequencies, indicating high levels of genotypic heterogeneity. Accumulating SNP positions were detected in genomic regions *bro-a*, *bro-b*, *bro-c*, *bro-d* and *hr1* to *hr6* (Figure 4a, middle and bottom). The homogeneity of isolate BmNPV-My was further confirmed by the mapping of reads against reference BmNPV-India (Figure 4b). Since the reference was not based on one of the sequenced BmNPV samples, the identification and determination of solely BmNPV-My specific SNP positions (*n* = 18; ƒ = 100%) was possible (Figure 4a, top). The My + De + Ja specific SNPs further supported the purity of BmNPV-My (*n* = 631; ƒ = 100%) representing the difference of all three sequenced samples to the reference. For isolate BmNPV-De the median frequencies of De + Ja (ƒ = 47.6%; *n* = 1113) and My + De + Ja (ƒ = 46.3%, *n* = 631) specific SNP positions were similar to the BmNPV-My reference-based analysis, supporting the assumption of two major genotypes. An increased genetic variability was also visible in *bro-a*, *bro-b*, *bro-c*, *bro-d* and *hr1* to *hr6* (Figure 4b). Based on both references, a drop of SNP frequencies below the medians was visible in regions around 5 kb, as well as 80 to 95 kb (Figure 4a,b). 

The above mentioned findings indicate that the isolates BmNPV-De and BmNPV-Ja are both highly similar in their genotypic composition and are mixtures of about equal amounts of two major BmNPV variants. On the contrary, the population structure of BmNPV-My appeared rather pure and homogenous, with almost no or few genetic variations, demonstrating the heterogeneous and homogenous diversity within BmNPV isolates from India.

### 3.2. Isolate Composition of BmBDV-Ja

From the sequenced BmNPV-De and BmNPV-Ja samples, only 77% and 35% of all reads, respectively, were identified as belonging to BmNPV (Table 2); therefore, the non-related reads were checked. Analyses were performed on the entity of de novo assembled contigs by blast analysis, which resulted in the findings of BmNPV contigs for BmNPV-De and -Ja, as well as the entire genome for BmNPV-My (Figure 3).

**Figure 5:**
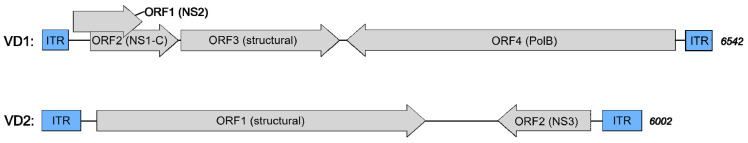
Bipartite linear ssDNA genome of BmBDV-Ja. The length of genome segments VD1 (**top**) and VD2 (**bottom**) are given in nucleotides (nt). Gray arrows indicate annotated ORFs and their orientations. Inverted terminal repeats (ITR) are marked in blue.

From sample BmNPV-Ja, both linear genome molecules (VD1 = 6542 nt and VD2 = 6002 nt) of a bidensovirus could be reconstructed and annotated. They were termed BmBDV-Ja VD1 and BmBDV-Ja VD2 (Figure 5). VD1 contains four putative ORFs (NS2, NS1-C, structural and PolB), whereas VD2 was found to encode two ORFs (structural and NS3) (Figure 5). 

**Figure 6:**
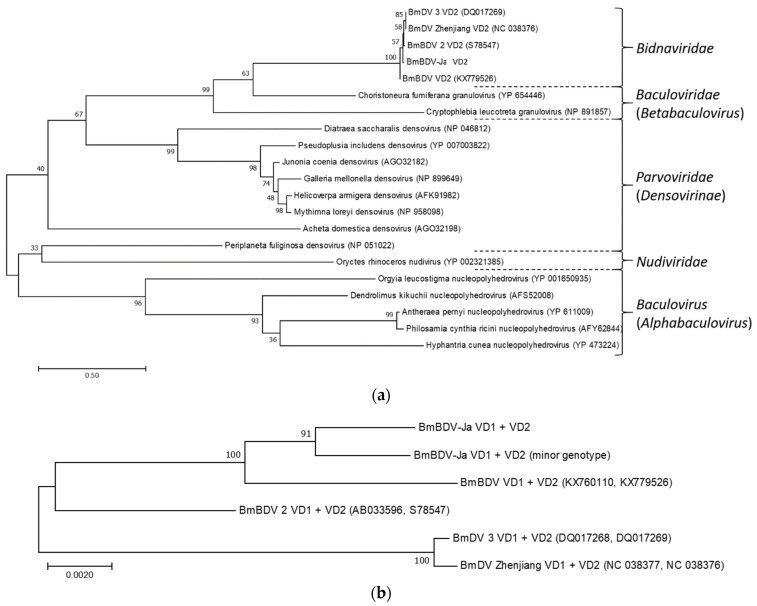
(**a**) Maximum likelihood tree based on the NS3 protein sequences and homologs in selected virus families [38]. (**b**) Maximum likelihood tree of the concatenated bipartite (VD1 and VD2) genome of Bombyx mori bidensoviruses. VD1, spacer and VD2 genome were used further for SNP detection analysis (see Figure 7). The BmBDV-Ja (minor genotype) sequence reflects the consensus sequence of BmBDV-Ja, including all alternative nucleotides with frequencies ƒ > 10% in variable SNP positions that were detected in the BmBDV-Ja based SNP analysis. BmBDV—Indian isolate; BmBDV 2—Yamanashi isolate; BmDV 3—China isolate; BmDV Zhejiang—Zhenjiang isolate. GenBank accession numbers are given in brackets behind virus names or abbreviations.

Both genome molecules were flanked by inverted terminal repeats. Based on the phylogeny of NS3, the bidensovirus gene of which homologs were found in other virus families, such as *Nudiviridae* and *Baculoviridae* [38,39], the virus was identified as Bombyx mori bidensovirus of the *Bidnaviridae* and was termed BmBDV-Ja (Figure 6a). To verify its taxonomic status in relation to previously identified and sequenced BmBDV isolates, the nucleotide sequences of the segments VD1 and VD2 were concatenated, followed by a whole genome alignment. For technical reasons, both genome segments were separated by an identical random 300 nt spacer (Figure S1). Upon alignment, the spacers of all sequences were perfectly aligned beneath each other, and therefore had no influence on the BmBDV phylogeny. According to the concatenated bipartite genome analysis, the BmBDV-Ja was most closely related to BmBDV (Indian isolate; KX760110 and KX779526) [21], with both building a common clade (Figure 6b). BmBDV 2 (Yamanashi isolate; AB033596 and S78547) [22] and the clade comprising BmDV 3 (China isolate; DQ017268 and DQ017269) [23] and BmDV Zhenjiang (Zhenjiang isolate; NC038377 and NC038376) were more distantly related (Figure 6b). The same analysis was repeated independently and separately for VD1, as well as VD2, but resulted in the same phylogenetic classification (Figure S3).

To determine any genotypic variance within the newly detected bidensovirus of the sequenced BmNPV samples, the concatenated BmBDV-Ja sequence was used as reference for SNP detection, similar to the BmNPV approach shown above. The reference was chosen due to its close relationship to the examined samples in order to reduce the number of detected variable SNP positions, and thus the complexity of the quantitative analysis. The reads of all sequenced samples were mapped to the concatenated BmBDV-Ja genome and the alignment files were used for the detection of variable SNP positions. In total, 163 variable SNP positions were found.

**Figure 7:**
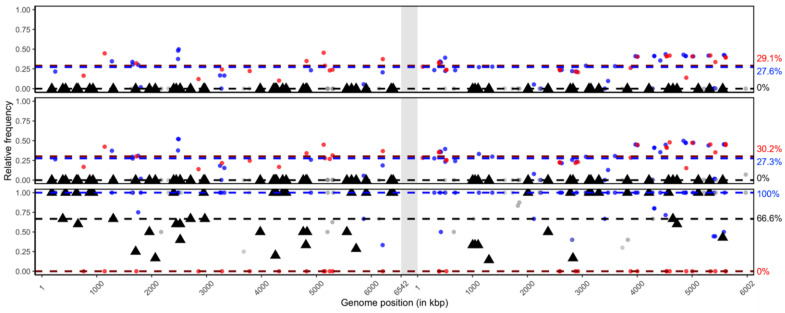
Common reference-based SNP detection method using BmBDV-Ja VD1 (left: 1 to 6542 nt) and BmBDV-Ja VD2 (right: 1 to 6002 nt), concatenated with a random 300 nt spacer (light grey vertical box). The entity of sequenced reads of the BmNPV-Ja (**top**), BmNPV-De (**middle**) and BmNPV-My (**bottom**) samples were used for mapping on the concatenated reference and the detection of bidensovirus reads. The SNP detection, specificity determination and frequency calculation were performed identically, as described for BmNPV (Figure 4). Refer to the Materials and Methods section for further details. Black triangles: solely BmBDV-My specific; red: BmBDV-De and BmBDV-Ja specific; blue: BmBDV-My, BmBDV-De and BmBDV-Ja specific; light grey dots: either BmBDV-My + BmBDV-De or BmBDV-My + BmBDV-Ja specific SNP positions. Median SNP frequencies are presented by black (=BmBDV-My), blue (=BmBDV-De and BmBDV-Ja) and red (=BmBDV-My, BmBDV-De and BmBDV-Ja) dashed lines. Medians are given to the right of the plot in corresponding colors.

The mapping of bidensovirus reads originating from the sequenced BmNPV-Ja and BmNPV-De samples was highly similar, with only minor differences in SNP frequencies (Figure 7, top and middle). Therefore, both bidensoviruses of the BmNPV-Ja and BmNPV-De sample were considered as extremely similar, visible by the overall SNP frequency patterns (Figure 7, top and middle). In both read alignments, the median frequencies of the My + De + Ja and De + Ja specific SNP positions were between ƒ = 29% to 30% and between ƒ = 27% to 28%, respectively (Figure 7, top and middle). Since the detected SNP frequencies were below 50%, it was concluded that BmBDV-Ja consensus sequence reflected the major present genotype in these sequenced samples. To analyze the presence of a possible second genotype, which was reflected by the alternative nucleotides in the detected SNP positions, a second consensus sequence was formed, which took variable SNP positions with frequencies above ƒ = 10% into account. This sequence was designated BmNPV-Ja (minor genotype) and was included into the phylogenetic studies (Figure 6b). The aim was to check whether the detected variance was due to an already described bidensovirus (for example, the Indian BmBDV isolate) which was not supported, since the consensus sequence of the putative BmBDV-Ja (minor genotype) was grouped closer to BmBDV-Ja (major genotype) than to any bidensovirus of this study (Figure 6b). 

The presence of a BmBDV within the BmNPV-My sample could be confirmed by the mapping of 275 and 283 reads to VD1 and VD2, respectively (Table 2). The low proportion of less than 0.1% BmBDV reads to the entire sequencing data indicated its minor presence in the BmNPV-My sample. Due to the low number of reads, the SNP analysis was not reliable, but it indicated that the present BmBDV isolate had major differences in SNP frequencies. In particular, this was visible in the My specific SNP positions (ƒ = 66.6%, *n* = 54) and the SNPs specific to My+De+Ja (ƒ = 100%, *n* = 53). Only the De+Ja specific SNP positions were identical to the reference (ƒ = 0%, *n* = 33) (Figure 7, bottom). This could indicate that the more geographically distant sample of BmNPV-My did contain more different BmBDV genotypes than those present in the BmNPV-De and BmNPV-Ja samples.

## 4. Discussion

Among the various diseases, grasserie caused by BmNPV is the most severe and contagious disease of *B. mori* that prevails in all rearing seasons and climatic regions, with varying prevalence [6]. In the seven BmNPV isolates from India described in this study, a certain homogeneity in the northern (BmNPV-Ka, BmNPV-Gh) and southern (BmNPV-An, BmNPV-My and BmNPV-Sa) samples of India was described on the basis of selected PCR markers. Only the partial gene marker of the BmNPV-De and BmNPV-Ja samples indicated significant differences to the other geographic isolates, and were therefore selected for further sequencing analyses. Since the Karnataka region, to which the city of Mysore belongs, is one of the main sericulture practicing areas in India, the BmNPV-My sample was also included for whole genome sequencing as tropical isolate. The National Silkworm Seed Organization (NSSO), located in Karnataka, supplies silkworm seeds to other sericulture practicing regions of India (http://www.csb.gov.in/, accessed on 12 May 2021), which could indicate why the geographic isolates from the North (BmNPV-Ka, BmNPV-Gh) and the South (BmNPV-An, BmNPV-My and BmNPV-Sa) were identical on the basis of the partial gene phylogeny.

BmNPV have been isolated and characterized from different parts of the world, highlighting the importance of BmNPV to the mass-rearing of *B. mori*. Thus far, the genome description of BmNPV isolates was mainly restricted to consensus sequences that reflected the majority nucleotide composition of an isolate [9,40]. New approaches in baculovirus genomics have aimed to decipher the genetic composition of isolates and allowed the differentiation and bioinformatic presentation of complex genotype mixtures [25,26,37,41]. Here, this approach was successfully transferred to an alphabaculovirus in the example of BmNPV. It demonstrated high sequence homogeneity or genetic purity in BmNPV-My, on the one hand, and levels of high heterogeneity, with the distinction of two major genotypes in BmNPV-De and BmNPV-Ja, on the other. This newly applied approach greatly helped in determining the genetic variation between different geographic samples, emphasizing the need to consider intra-isolate variation before creating consensus sequences, not only for BmNPV but also for baculoviruses in general.

In previous studies focusing on the genetic diversity of BmNPV and BomaNPV, both of which belong to the *Bombyx mori nucleopolyhedrovirus* species [13,15], differentiation between isolates was based on genome consensus sequences [40]. By aligning the genome sequences of BomaNPV-S1, BmNPV-Cubic, BmNPV-Guangxi, BmNPV-India, BmNPV-T3 and BmNPV-Zhejiang, a total of 832 variable positions were reported [40], representing about half the number of variable positions that were found for the Indian samples from Mysore (BmNPV-My), Dehradun (BmNPV-De) and Jammu (BmNPV-Ja) by using a more in-depth approach focusing on intra-isolate specific variation. In total, about 1700 variable SNP positions were detected for only three newly sequenced isolates from North and South India of this study, but the number was influenced by repeat regions, such as *hrs* regions and *bro* genes, to which reads of the sequencing data misaligned partially; this is visible by the column-like arrangement of SNPs in these genome areas.

The most striking discovery was the detection of two major genotypes within the geographic isolates BmNPV-De and BmNPV-Ja, both originating from North India, and the overall resembling genetic composition of both isolates. Although specific genotypes of large dsDNA baculovirus genomes are often difficult to identify in short-read sequencing data, the presence of these two major genotypes in both isolates was the most reasonable explanation. Therefore, BmNPV-Ja and BmNPV-De were considered as mixed isolates, with certain degree of heterogeneity that was reflected by the occurring SNP frequencies deviating from the median frequencies.

The terminology of homogenous, heterogeneous and mixed isolates was first introduced for the Cydia pomonella granulovirus (CpGV), a baculovirus of the genus *Betabaculovirus* [25,42]. Similar to BmNPV-My, the Mexican and Canadian isolates CpGV-M and CpGV-S, respectively, were identified as highly pure in their genotype composition, since variable SNP positions were missing [25,43]. The English isolate CpGV-E2 was identified as highly heterogeneous, since a clear demarcation of a certain genotype was not possible ([25,28], whereas other CpGV isolates appeared to be mixtures of rather pure genotypes [25,28]. Although the SNP patterns of BmNPV-De and BmNPV-Ja suggest that both isolates are mixtures of two main variants, the underlying major genotypes of BmNPV-De and BmNPV-Ja were unknown, which was reflected by SNP positions being specific for none of the BmNPV isolates included in this study. Another interesting observation was the detection of possible regions of recombination between these two major genotypes at around 5 kb, as well as 80 to 95 kb, where a drop in the alternative nucleotide frequencies, and therefore an increase of the reference nucleotide frequency, was noted, respectively. This hint for genome recombination based on SNP analysis was also previously described for isolate CpGV-R5 in multiple genome regions, and for CpGV-WW [25,28,43]. Besides the mixed character of BmNPV-De and BmNPV-Ja, a certain level of an overall genetic heterogeneity was also observed, underlining the complex genetic diversity within these BmNPV isolates. The complexity of alphabaculovirus genome populations within single isolates was further reflected by sequencing approaches on AcMNPV-WP10, as well as an Argentinian isolate of the Spodoptera frugiperda multiple nucleopolyhedrovirus (SfMNPV-ARG-M) [24,26]. For SfMNPV-ARG-M, a number of 704 highly variable SNP positions were detected [26], describing this isolate as heterogeneous without a differentiation of major or minor genotypes. For AcMNPV-WP10, an ultra-deep sequencing approach was followed, where an average read depth of >124 k was achieved, which resulted in the detection of 3242 SNP positions, most of which with nucleotide frequencies ranging between 10% < ƒ < 50% [24]. Since both *Autographa californica multiple nucleopolyhedrovirus* and *Bombyx mori nucleopolyhedrovirus* species are closely related to each other, it is not clear whether a high genetic diversity within both alphabaculoviruses reflects the genome variability within the entire genus, or only within the taxonomic BmNPV and AcMNPV clade. 

In addition to the identification of BmNPV, the BmNPV-De and BmNPV-Ja samples further contained a significant amount of BmBDV belonging to the *Bidnaviridae* family, whose family members were exclusively isolated from *B. mori* [38]. The co-purification of BmBDV in the samples was unexpected, since the applied purification methods were specifically applied for the large OBs of BmNPV, not the 18 to 26 nm small and icosahedral particles of bidensoviruses. Therefore, the purification procedure used in this study has to be considered as qualitative but not quantitative for BmBDV, and most likely influenced the recovery BmBDV from the samples. Thus, the observed amounts of BmNPV and BmBDV reads may not reflect the actual ratio of both viruses in the insects.

Similar to the findings for BmNPV, the genotypic composition of BmBDV appeared to be mixed within the BmNPV-Ja and BmNPV-De samples, although at different ratios and possibly homogenous for the BmNPV-My sample. For the latter, a conclusion was difficult to draw due to the low number of BmBDV reads that led to ambiguous results.

The different ratios of BmNPV and BmBDV within single samples could be explained by the propagation and creation of the BmNPV OB stocks, which were based on pooling several deceased larvae. Here, individual larvae could have been infected at different degrees by either one or both virus(es), resulting in the different virus ratios. Whether the handling of the OB stocks also had an impact on the similar genetic composition of the BmNPV-De and BmNPV-Ja samples cannot be conclusively clarified. 

The SNP-based analysis benefited from the concatenation of both genome fragments that allowed a whole genome comparison. According to the SNP-based approach, the VD1 and VD2 fragments followed similar patterns in their genotype composition, indicating that both genome fragments might undergo same selection pressure and that the BmBDV, which was found in the BmNPV-Ja and BmNPV-Ja samples, were mixtures of two main genotypes in a 70% to 30% ratio. Since the major genotype was reflected by the newly assembled consensus sequence, a minor genotype was extracted that comprised all alternative SNPs, with a frequency of ƒ > 10%. This was important to confirm that the potential major and minor genotypes were not similar or identical to one of the previously described bidensovirus sequences [23,38,44].

The present study revealed the genetic complexity and genotype composition within BmNPV and BmBDV isolates, both significant pathogens of the commercial important silkworm, collected from different geographical regions of India. The presented findings have broad implications for our understanding of the genetic diversity and evolution of BmNPV and co-occurring BmBDV isolates in a broader context, and expand our knowledge about the genetic variation within the *Baculoviridae* and *Bidnaviridae* family. Our results underline the importance of considering field collected samples of BmNPV as genotype populations, the compositions of which need to be carefully investigated for associated viruses, such as BmBDV. Deep sequencing combined with SNP analyses provides powerful tools for studying such samples, and will contribute to develop suitable management strategies for protecting the economically valuable silkworm from BmNPV and BmBDV pathogens. 

## Figures and Tables

**Table 1:** Geographic origin and partial gene sequences of BmNPV isolates (for details, see [6]).

Isolate	City, Region or State	GenBank Accession No.		
		*Polh*	*lef-8*	*lef-9*
BmNPV-An	Anantapur, Andhra Pradesh	MK415967	MK415970	MK415956
BmNPV-De	Dehradun, Uttarakhand	MK415968	MK415975	MK415957
BmNPV-Gh	Ghumarwin, Himachal Pradesh	MK415966	MK415974	MK415958
BmNPV-Ja	Jammu, Jammu and Kashmir	MK415964	MK415976	MK415959
BmNPV-Ka	Kashmir, Jammu and Kashmir	MK415969	MK415973	MK415960
BmNPV-My	Mysore, Karnataka	MK415963	MK415971	MK415961
BmNPV-Sa	Salem, Tamil Nadu	MK415965	MK415972	MK415962

**Table 2:** Number of reads that were generated and used for sequence analysis in this study. SD: standard deviation and RPKM: reads per kilo base per million mapped reads.

OB Stock	Total Reads Sequenced	Phred ≥ 30	No. Reads Mapping to BmNPV * (%)	BmNPV Mean Read Depth ± SD *	No. Reads Mapping to BmBDV-Ja ** VD1 (%)/VD2 (%)	BmBDV-Ja Mean Read Depth ± SD **	RPKM of VD1/VD2	Ratio of VD1/VD2 (~Fragment Ratio)
My	528,646	491,996	468,827 (95%)	507 ± 70	275 (<0.1%)/ 283 (<0.1%)	5 ± 2 6 ± 4	75,334/ 84,500	0.89 (~9:10)
De	946,752	855,476	659,811 (77%)	661 ± 126	63,593 (7.4%)/ 97,297(11.4%)	453 ± 89 696 ± 127	63,593/ 97,297	0.65 (~2:3)
Ja	797,784	704,544	248,770 (35%) ***	270 ± 39	62,277 (8.8%)/ 98,732 (14.0%)	689 ± 116 1109 ± 192	62,277/ 98,732	0.63 (~3:5)

* Mapping was performed using BmNPV-My (MW842985) as a reference sequence. ** Both genome molecules BmNPV-Ja VD1 (MW842986) and VD2 (MW842987) were created in this study and used for mapping of reads (see Figure S3). *** Sample contaminated with *Serratia marcescens.*

## Data Availability

Sequencing data are available under BioProject ID PRJNA724724.

## Supplementary Materials

Supplementary materials can be found at https://www.mdpi.com/article/10.3390/v13050901/s1.
