# Supplementary material for: Patterns in Genotype Composition of Indian Isolates of the Bombyx mori Nucleopolyhedrovirus and Bombyx mori Bidensovirus
Source: Viruses. 2021 May 13;13(5):901. doi: 10.3390/v13050901 (PMC8152266; doi:10.3390/v13050901)
Supplement: Supplementary file 1 [file viruses-13-00901-s001.zip › viruses-1186768-supplementary.pdf]

viruses

MDPI

Supplementary Material:

1–50 nt	CGATCCACGAACATCCACCTGATTACGAGTGTCGTTAGGGGCTTATCAGC
51–100 nt	TCCATGGGTTAATCTAGGTATAAGTCACGAAGCGACACCGAGAAGAGGCT
101–150 nt	CAAGCCCCCCAAATTCCATAAAGCTACGGAAGGGGACACCCTGGGCAGCG
151–200 nt	ATGCGCAGGGTAGTCCGTTTTATATCATACGCGTCCGCGGTTAATATACT
201–250 nt	ATTTGCATTTTCCACAACCTGTTGCATAGCGCGACGGCAGGCCCTTAGTA
251–300 nt	CAGTTAGAGGAGATACAAGCCTGCACCCTGTTGCCCTACCATCTGATACG

Figure S1. Random 300 nt linker sequence that was used for concatenating VD1 and VD2 of BmBDV-Ja.

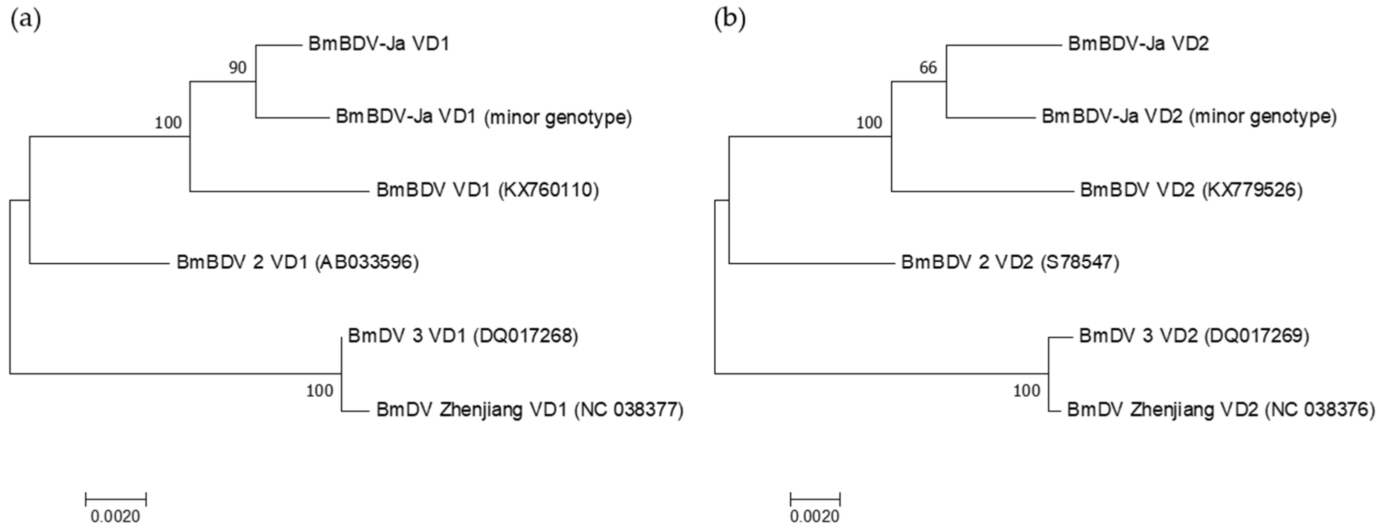

Figure S2. Maximum likelihood tree of the (**a**) VD1 and (**b**) VD2 genome molecules of BmBDV-Ja in comaprison to BmBDV, BmBDV2, BmDV 3 and BmDV Zhejiang (for details see Figure 6). The BmBDV-Ja (minor genotype) consensus sequence included all variable SNPs that were detected in the BmBDV-Ja based SNP analysis with a frequency *f* > 10%.

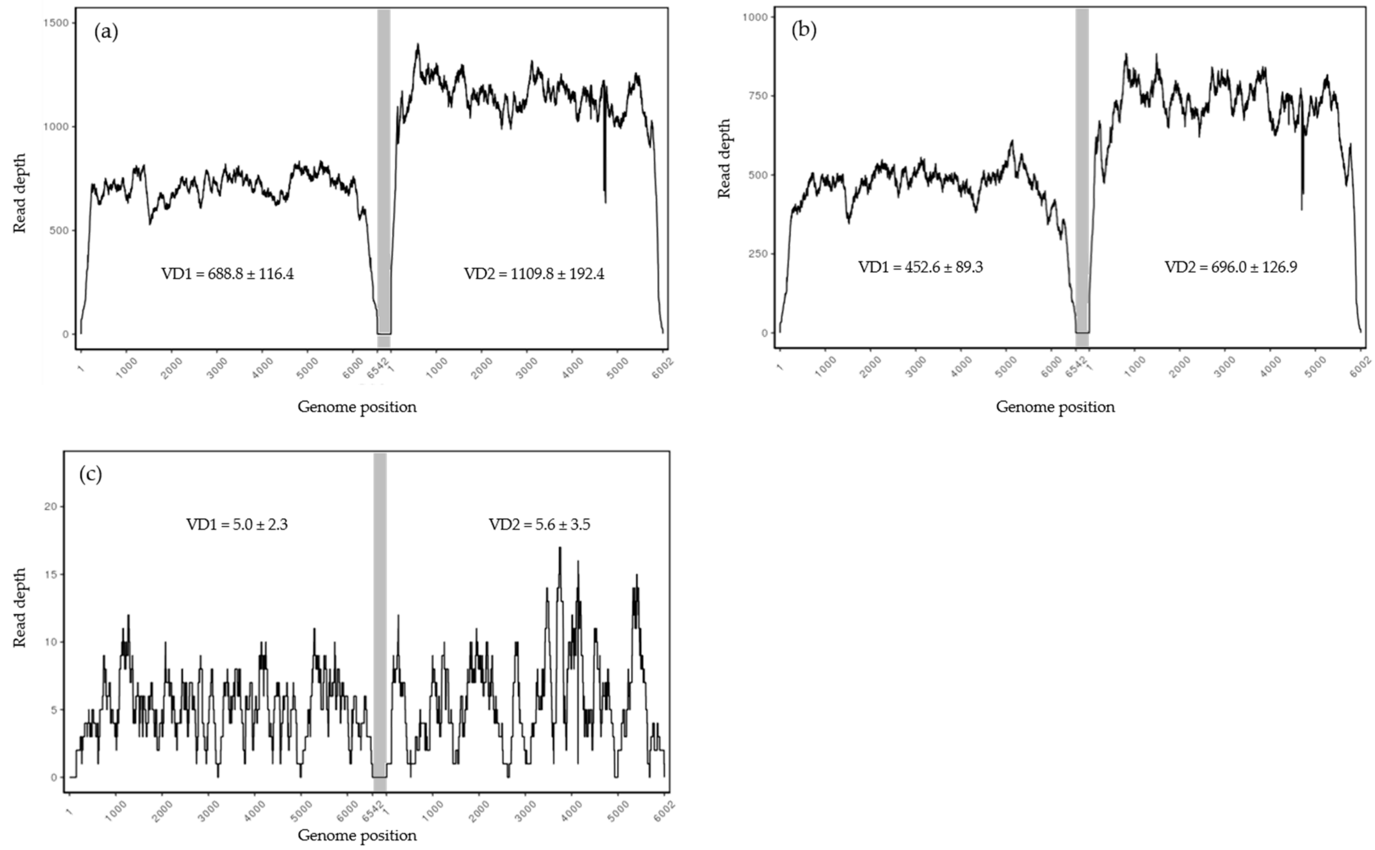

Figure S3. Read depth of the BmBDV alignments created from the sequenced (**a**) BmNPV-My, (**b**) BmNPV-De and (**c**) BmBDV-Ja samples. Both genome molecules (VD1 and VD2) of the bipartite BmBDV-Ja genome were concatenated by a 300 nt random spacer sequence (Figure S1) marked by a vertical grey box, resulting in a single genome molecule that was used as reference sequence. From the aligned reads, the mean read depth +/- standard deviation were calculated separately for VD1 and VD2.

Table S1. Open reading frames (ORF), features and nucleotide (nt) and amino acid (aa) similarity in comparison to the BmNPV-India (JQ991010) isolate.

ORF	Feature	Start		End	Length (nt)	nt identity (%)	aa identity (%)
bm1	*polh*	1	>	738	738	100	100
bm2	*orf1629*	768	<	2411	1644	98.97	99.1
bm3	*pk1*	2410	>	3240	831	99.28	99.3
bm4	*hypothetical protein*	3266	<	4288	1023	99.61	100
bm5	*hypothetical protein*	4630	<	5625	996	99.6	99.7
bm6	*lef-1*	5505	<	6317	813	99.88	100
bm7	*egt*	6432	>	7952	1521	99.41	98.4
bm8	*odv-e26*	8094	>	8783	690	99.13	98.7
bm9	*hypothetical protein*	8752	>	9384	633	99.37	99.5
bm10	*hypothetical protein*	9414	<	10484	1071	99.16	98.6
bm11	*hypothetical protein*	10486	>	10818	333	99.4	100
bm12	*airf-1*	11001	<	12347	1347	99.03	98.7
bm13	*pif-2*	12384	>	13532	1149	99.65	99.5
bm14	*f-protein*	13635	>	15656	2022	99.95	99.9
bm15	*pkip*	15687	<	16196	510	99.22	100
bm16	*dbp*	16236	<	17192	957	99.58	99.7
bm17	*hypothetical protein*	17268	>	17657	390	100	100
bm18	*iap1*	17659	>	18534	876	99.2	99.3
bm19	*lef-6*	18539	>	19060	522	100	100
bm20	*hypothetical protein*	19183	<	19398	216	100	100
bm21	*hypothetical protein*	19454	<	20872	1419	99.72	99.6
bm22	*bro-a*	20907	<	21878	972	94.66	93.6
bm23	*sod*	22056	>	22511	456	100	100
	hr1	22607		23482			
bm24	*fgf*	23540	>	24088	549	99.64	100
	hr2	24148		24336			
bm25	*hypothetical protein*	24401	<	25048	648	100	100
bm26	*ubiquitin*	25069	>	25302	234	99.57	100
bm27	*39k*	25352	<	26188	837	100	100
bm28	*lef-11*	26182	<	26520	339	100	100
bm29	*bv-e31*	26483	<	27136	654	99.54	100
bm30	*p43*	27204	<	28292	1089	99.63	99.4
bm31	*p47*	28300	<	29499	1200	99.75	99.7
bm32	*lef-12*	29504	>	30037	534	99.06	97.2
bm33	*gta*	30112	>	31632	1521	99.34	99.2
bm34	*hypothetical protein*	31646	>	31882	237	100	100
bm35	*hypothetical protein*	31863	>	32258	396	98.74	100
bm36	*hypothetical protein*	32260	>	32844	585	98.97	99
bm37	*odv-e66*	32829	>	34955	2127	99.86	99.9

ORF	Feature	Start		End	Length (nt)	nt identity (%)	aa identity (%)
bm38	*ets*	35055	<	35324	270	99.63	98.9
bm39	*lef-8*	35574	<	38177	2604	99.77	100
bm40	*hypothetical protein*	38234	>	39193	960	99.69	100
bm41	*hypothetical protein*	39184	<	39768	585	98.97	97.9
bm42	*hypothetical protein*	39770	>	40189	420	100	100
bm43	*lef-10*	40186	>	40422	237	100	100
bm44	*vp1054*	40280	>	41377	1098	100	100
bm45	*hypothetical protein*	41459	>	41680	222	94.02	93.5
bm46	*hypothetical protein*	41682	>	41936	255	100	100
bm47	*hypothetical protein*	42190	>	42675	486	99.59	99.4
bm48	*hypothetical protein*	42690	<	43205	516	100	100
bm49	*ac60*	43217	<	43465	249	100	100
bm50	*fp25*	43611	<	44255	645	100	100
bm51	*lef-9*	44359	>	45831	1473	99.86	100
bm52	*hypothetical protein*	45892	>	46359	468	99.57	100
bm53	*gp37*	46433	<	47317	885	98.87	97.6
bm54	*dnapol*	47447	<	50413	2967	99.73	100
bm55	*hypothetical protein*	50422	>	52839	2418	99.83	99.9
bm56	*lef-3*	52842	<	53999	1158	100	100
bm57	*odv-nc42*	54018	>	54422	405	100	100
bm58	*hypothetical protein*	54400	>	55188	789	100	100
bm59	*iap2*	55355	>	56104	750	99.87	100
bm60	*hypothetical protein*	56163	>	56345	183	100	100
bm61	*hypothetical protein*	56356	<	56655	300	100	100
bm62	*hypothetical protein*	56652	<	57461	810	99.88	100
bm63	*hypothetical protein*	57479	<	57880	402	99	99.2
bm64	*hypothetical protein*	57899	<	58156	258	100	100
bm65	*vlf-1*	58172	<	59326	1155	98.1	97.9
bm66	*hypothetical protein*	59332	<	59664	333	99.7	99.1
bm67	*hypothetical protein*	59667	<	59981	315	100	100
bm68	*gp41*	59984	<	61189	1206	99.67	99.8
bm69	*hypothetical protein*	61179	<	61886	708	99.72	100
bm70	*hypothetical protein*	61729	<	62277	549	100	100
bm71	*p95*	62243	>	64756	2514	99.64	99.6
	hr3	64778		66264			
bm72	*vp15*	66308	>	66688	381	99.48	100
bm73	*cg30*	66693	<	67493	801	99.25	98.9
bm74	*vp39*	67496	<	68542	1047	100	100
bm75	*lef-4*	68561	>	69958	1398	99.64	100
bm76	*hypothetical protein*	69955	<	70416	462	99.78	99.3
bm77	*p33*	70452	<	71231	780	99.62	100
bm78	*hypothetical protein*	71230	>	71715	486	99.59	100

ORF	Feature	Start		End	Length (nt)	nt identity (%)	aa identity (%)
bm79	*odv-e25*	71724	>	72410	687	100	100
bm80	*dna helicase*	72449	<	76117	3669	99.78	100
bm81	*odv-e28*	76104	>	76625	522	98.28	97.7
bm82	*bro-b*	76740	>	77465	726	n.a.	n.a.
bm83	*bro-c*	77524	<	78522	999	93.29	92.5
bm84	*38k*	78665	<	79627	963	99.58	99.4
bm85	*lef-5*	79562	<	80359	798	99.62	99.6
bm86	*p6.9*	80356	<	80553	198	100	100
bm87	*p40*	80595	<	81686	1092	99.91	100
bm88	*p12*	81706	<	82083	378	100	100
bm89	*p45*	82064	<	83227	1164	99.48	99.7
bm90	*vp80*	83253	<	85337	2085	99.62	99
bm91	*he65*	85360	<	86229	870	99.43	99.3
	hr4L	86250		86846			
bm92	*hypothetical protein*	86863	<	87609	747	99.33	98.8
bm93	*hypothetical protein*	87610	<	87927	318	100	100
bm94	*hypothetical protein*	87942	<	89117	1176	99.4	99.5
bm95	*hypothetical protein*	89141	<	89320	180	100	100
bm96	*hypothetical protein*	89369	<	89572	204	100	100
	hr4R	89715		90241			
bm97	*hypothetical protein*	90246	<	91520	1275	99.76	99.7
bm98	*pif-3*	92155	<	91535	614	99.84	99.5
bm99	*hypothetical protein*	92333	<	92163	185	94.05	89.5
bm100	*hypothetical protein*	92269	<	92556	288	99.65	100
bm101	*pif-1*	92688	<	94271	1584	99.62	99.4
bm102	*hypothetical protein*	94279	<	94527	249	100	100
bm103	*hypothetical protein*	94630	<	94803	174	100	100
bm104	*hypothetical protein*	94696	<	94881	186	100	100
bm105	*gcn2*	94915	<	95592	678	99.56	99.1
bm106	*hypothetical protein*	95776	<	96510	735	99.05	98.8
bm107	*lef-7*	96529	<	97212	684	98.68	97.8
bm108	*chitinase*	97202	<	98857	1656	99.64	99.6
bm109	*v-cath*	98904	<	99875	972	99.9	100
bm110	*gp64*	99993	<	101585	1593	100	100
bm111	*p24*	101713	<	102300	588	99.83	100
bm112	*gp16*	102331	<	102651	321	100	100
bm113	*pp34*	102713	<	103660	948	99.79	99.7
bm114	*hypothetical protein*	103663	<	104325	663	99.55	99.5
bm115	*alkexo*	104353	<	105615	1263	99.52	99.5
bm116	*p35*	106082	<	106981	900	100	100
	hr5	107030		107914			
bm117	*p26*	107971	<	108693	723	100	100

ORF	Feature	Start		End	Length (nt)	nt identity (%)	aa identity (%)
bm118	*p10*	108766	<	108978	213	99.53	100
bm119	*p74*	109053	<	110990	1938	99.79	99.5
bm120	*me53*	111187	<	112542	1356	99.48	99.1
bm121	*hypothetical protein*	112688	<	112891	204	100	100
bm122	*ie-0*	112819	<	113604	786	99.62	100
bm123	*odv-nc50*	113619	<	115049	1431	99.86	99.8
bm124	*odv-e18*	115060	<	115368	309	100	100
bm125	*odv-ec27*	115383	<	116255	873	99.89	100
bm126	*hypothetical protein*	116270	<	116557	288	100	100
bm127	*hypothetical protein*	116552	<	117157	606	99.67	100
bm128	*ie-1*	117223	<	118977	1755	99.83	100
bm129	*odv-e56*	119093	<	120223	1131	99.29	98.7
bm130	*hypothetical protein*	120252	<	120572	321	100	100
bm131	*hypothetical protein*	120541	<	120858	318	100	100
bm132	*ie-2*	120891	<	122168	1278	98.51	98.4
bm133	*pe38*	122654	<	123583	930	100	100
bm134	*hypothetical protein*	123688	<	123924	237	99.58	100
	hr6	123929		124392			
bm135	*ptp*	124403	<	124909	507	99.21	98.8
bm136	*bro-d*	124906	<	125952	1047	94.57	92.6
bm137	*hypothetical protein*	126026	<	126481	456	99.12	99.3
bm138	*hypothetical protein*	126510	<	126839	330	99.39	99.1
bm139	*lef-2*	126820	<	127452	633	99.53	99.5
